# Effects of Visible and UV Illumination on Dimensional Accuracy and Surface Roughness in Dual-Color Volumetric Additive Manufacturing (VAM)

**DOI:** 10.3390/ma19071285

**Published:** 2026-03-24

**Authors:** Seyyed Kaveh Hedayati, Hossein Safari Mozajin, Azar Najafi Tireh Shabankareh, Kristoffer Almdal, Yi Yang, Aminul Islam

**Affiliations:** 1 Department of Civil and Mechanical Engineering, Technical University of Denmark, 2800 Kgs. Lyngby, Denmark; 2 Department of Chemistry, Technical University of Denmark, 2800 Kgs. Lyngby, Denmark

**Keywords:** dimensional accuracy, surface analysis, volumetric additive manufacturing (VAM), photopolymer

## Abstract

Volumetric additive manufacturing (VAM) enables layerless and fast printing within seconds. However, print quality remains highly sensitive to the delivered energy. In this study, the effects of visible (460 nm) and ultraviolet (385 nm) projector power were evaluated in a dual-color VAM setup with a CQ/EDAB initiated TEGDMA/BisGMA resin with an *o*-Cl-HABI inhibitor. Cubes ($6\times 6\times 6.7\;{\mathrm{mm}}^{3}$) were printed under controlled visible and ultraviolet power and exposure times, then evaluated using in situ shadowgraphy, three-dimensional metrology, and confocal microscopy. Higher visible power reduced the polymerization initiation time, but increasing the visible dose rapidly led to over-polymerization, resulting in dimensional growth, corner rounding, and increased surface roughness ($R_{a}$). The lowest lateral variation was observed at the shortest exposure times, with a maximum error of 1.8%. Ultraviolet illumination did not significantly change initiation time or reduce over-polymerization within the tested intensities and inhibitor concentration ranges. Surface evaluations revealed a periodic line texture with a pattern pitch of approximately 25 μm. By shifting the focal plane and using a low-resolution projector, the pattern pitch increased to about 150 μm. These values were aligned with the ${\mathrm{MTF}}_{50}$ spatial frequencies of each projector at different defocus positions. This study provides useful guidelines for adjusting intensity to achieve high-fidelity VAM printed parts.

## 1. Introduction

Fabrication of complex geometries directly from digital models was enabled by Additive manufacturing (AM). AM increasingly used to shorten design to production cycles across different industries [1,2]. However, most established AM processes, such as digital light processing (DLP) and Stereolithography (SLA), remain layer-based approaches, which can impose trade-offs between build rate, directional mechanical performance, and surface characteristics due to the conversion of digital models into cross-sectional layers [3]. The development of volumetric printing opened the opportunity to separate fabrication time from layer count while reducing surface artifacts, but also highlighted that both optical delivery and resin reaction must be carefully controlled to maintain precision [3,4]. Volumetric additive manufacturing (VAM) addresses these limitations by curing a three-dimensional shape within a resin volume using a programmed light dose distribution, rather than sequentially printing layers [3,4].

In VAM, the object is defined as a set of dynamic projections that generate a three-dimensional (3D) energy dosage distribution field (EDDF) within a rotating build volume; polymerization occurs where the EDDF exceeds a resin conversion threshold. Since the surrounding uncured resin supports the part during exposure, VAM can fabricate complex overhangs and negative structures without dedicated support structures [4,5].

Several VAM techniques have been demonstrated within past few years. Early “additive light superposition” approaches used intersecting, holographically shaped beams to trigger polymerization only where the combined dose exceeded threshold [6]. Tomographic VAM, also known as computed axial lithography (CAL), generalized this concept by projecting a sequence of 2D patterns [7]. Print fidelity was improved via advances in reconstruction strategies, optical design, and delivered dose optimization [8,9]. Other methods, such as Xolography and latent-image VAM, enhanced spatial control by restricting polymerization to beam intersections via two-color activation or by separating exposure from development to expand process control and minimize artifacts [10,11].

Achieving dimensional accuracy in VAM remains challenging since the intended EDDF can be distorted by optical blur, refraction, scattering in filled or heterogeneous resins, and non-linear resin kinetics [3,4,5]. Computational solutions such as object-space optimization can compensate for systematic dose errors and improve geometric fidelity [12]. Correcting ray distortion from the print container, can further reduce reconstruction artifacts [13], and in situ 3D metrology methods also enabled monitoring of the geometry formation during the printing process [14].

The printable materials are expanding beyond conventional acrylate resins to include thiol-ene networks, hydrogels, bioresins, and even inorganic precursors for glass and ceramic processing [5,15,16,17]. Resin formulation and photochemistry are critical to establish the threshold behavior required in this process. Mechanistic analyses underscore the roles of photoinitiator absorption, inhibitor depletion, and radical termination in shaping the conversion nonlinearity that separates “in-part” and “out-of-part” voxels [4]. Multi-wavelength strategies are particularly favorable since they can provide additional degrees of freedom to control the polymerization process. As a showcase, Dual-color VAM has been used to regulate spatial stiffness within the printed part [18], and binary photoinhibition can generate negative illumination to control over-polymerization and sharpen printed features [19]. Related dual-wavelength initiation-inhibition procedures and volumetric inhibition further describe how spectral selectivity can improve process stability [20,21].

Projection powers (intensities) alter not only dose accumulation but also the balance between initiation and inhibition pathways, thereby shifting the effective threshold and influencing surface formation and dimensional accuracy, especially in dual-wavelength systems [3,4,20]. As mentioned, dual-color VAM printing has been used to modulate crosslinking density and fabricate negative features locally. However, experimental studies that isolate Vis and UV output power and quantify their effects on polymerization initiation, shape accuracy, and surface roughness remain limited. In this study, we evaluate how the controlled variable, Vis and UV illumination intensity, influences the process. Using a representative resin formulated with a UV-responsive inhibitor, we isolate the specific effects of each wavelength on the final properties of the printed cubes. We aim to establish direct links between the projected optical dose and print characteristics, including polymerization initiation time, dimensional fidelity, and surface roughness. The effect of UV light, which activates the inhibitor in the studied resin, was also assessed. Furthermore, we correlate the optical resolution of the projection system with the surface textures. The findings present guidelines for tuning dual-color illumination to improve accuracy and reproducibly fabricate high-fidelity parts.

## 2. Materials and Methods

### 2.1. Materials and Resin Formulation

The photosensitive resin was prepared by mixing triethylene glycol dimethacrylate (TEGDMA, Sigma–Aldrich, Darmstadt, Germany) and bisphenol A glycerolate dimethacrylate (bisGMA, Sigma–Aldrich) at a 2:1 weight ratio. Camphorquinone (CQ, Sigma–Aldrich) and ethyl 4-dimethylaminobenzoate (EDAB, Sigma–Aldrich) were added as the photoinitiator and co-initiator at concentrations of 0.1 wt.% and 0.25 wt.%, respectively. In addition, 2,2^′^-Bis(2-chlorophenyl)-4,4^′^,5,5^′^-tetraphenyl-1,2^′^-biimidazole (o-Cl-HABI, TCI Europe, Zwijndrecht, Belgium) was dissolved in tetrahydrofuran (THF, Fisher Scientific, Waltham, MA, USA) at 28 wt.% and subsequently added to the resin as an inhibitor at a final concentration of 0.2 wt.%. All chemicals were used as received without further purification.

### 2.2. VAM Setup

**Figure 1:**
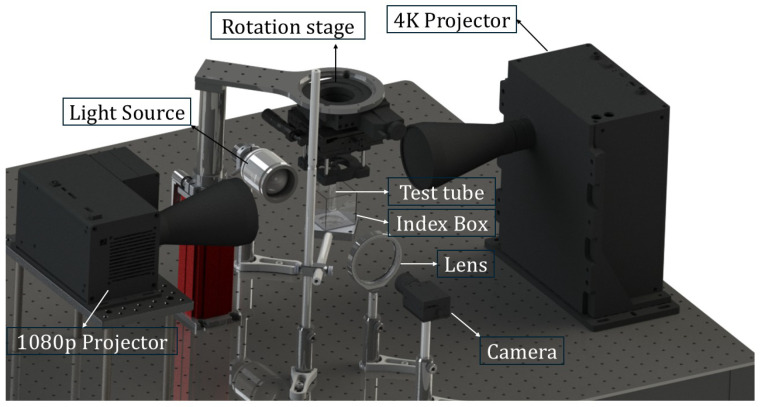
Rendered CAD representation of the custom-built VAM setup with both 4K and 1080P projectors.

A custom-made VAM setup was built using two dual-color projectors: a 4K and a 1080P DLP projector (TVP07, Xiamen Zhisen, Xiamen, Fujian, China) (Figure 1). The 4K DLP projector provides a focal-plane area of $32.4\times 57.6\;{\mathrm{mm}}^{2}$ with a nominal pixel size of 15 μm, capable of simultaneous modulation of blue (460 nm) and UV (385 nm) illumination. The 1080P projector had the same focal-plane area ($32.4\times 57.6\;{\mathrm{mm}}^{2}$) but a larger nominal pixel size of 30 μm, and supported modulation at 460 nm and 365 nm. The emission spectra of both projectors were characterized using a CCS100 spectrometer (Thorlabs, Mölndal, Sweden). For VAM printing, the tube was suspended on a rotating stage. The 4K projector focal plane was positioned 6 mm off-center relative to the tube axis, whereas the 1080P projector focal plane was concentrically aligned with the tube axis. In situ monitoring of the printing process was performed using a 4f imaging configuration with a CMOS camera (GS3-U3-51S5M-C, FLIR Grasshopper, Täby, Sweden) mounted perpendicular to the projection direction.

### 2.3. VAM Printing Procedure and Process Parameters

The influence of illumination on surface quality and dimensional accuracy was evaluated by fabricating cubic specimens with nominal dimensions of 6 × 6 × 6.7 mm^3^. Printing was performed in cylindrical glass test tubes (outer diameter 15.5 mm). To minimize optical distortion at the curved glass interface, the test tubes were immersed in an index-matching liquid formulated from the same resin (TEGDMA:bisGMA = 2:1, *w*/*w*) without any initiator.

**Figure A1:**
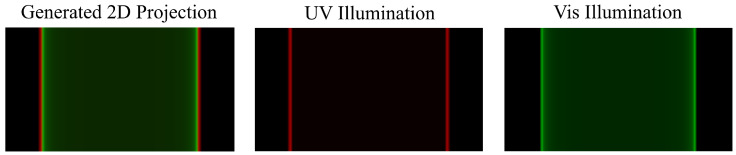
Representative 2D projection patterns used for VAM printings. The image shows an example of Vis (Green) and UV (Red) projection frames and a combined visualization.

All specimens (n = 3 for each condition) were printed using 360 projections at a projection rate of 10 fps. Three projection times were investigated in combination with three visible-light power settings (40%, 60%, and 80%) and two UV power settings (30% and 60%). The visible power levels corresponded to irradiances of 28, 48, and 62 mW cm^−2^, respectively, while the UV power levels were 24 and 37 mW cm^−2^. Representative examples of the 2D projection patterns used for the Vis and UV illumination channels are provided in Appendix A (Figure A1).

### 2.4. Post-Processing

**Figure A3:**
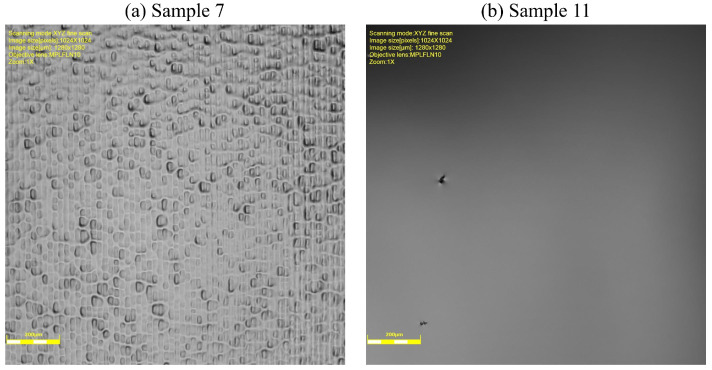
Representative confocal microscopy surface scans from the PP DOE study showing (**a**) the best and (**b**) the worst qualitative surface outcomes. (Scale bar: 200 μm).

To facilitate specimen release after printing, test tubes were heated up to 60 °C. Cured parts were carefully taken out and immersed in heated 2-propanol (IPA, VWR BDH Chemicals) for 10 min to dissolve any residual uncured resin. Several rinsing steps with fresh IPA were also performed at the end to complete surface and edge cleaning. Then, samples were allowed to dry in the ambient before post-curing. Final curing was done using 405 nm and 470 nm LED light sources (Solis series, Thorlabs) for 30 min under a nitrogen atmosphere to suppress oxygen inhibition and obtain non-sticky (tack-free) surfaces. The post-processing (PP) procedure was selected experimentally by monitoring a limited set of key parameters and conducting a brief design of experiments (DOE) study to ensure repeatability and minimize surface defects across all printed samples, as described in Appendix A (Table A1 and Figure A3). Later on, the selected PP procedure was fixed and applied identically to all specimens.

### 2.5. Surface Topography and Roughness Measurement

Surface characterization of the printed cube faces (one sample per condition) was performed using a confocal laser scanning microscope (Olympus LEXT OLS4100, Tokyo, Japan) at 10× magnification. Data were acquired using the Olympus LEXT OLS4100 software (v3.1.10) and further processed in MountainsMap (v9.3). Linear roughness was measured from ten equally spaced vertical line profiles aligned with the rotational axis of the test tube. Each surface profile was filtered with an S-filter (nesting index $\lambda_{s}=2.5\;\mu m$) to reduce measurement noise and an L-filter (cut-off wavelength $\lambda_{c}=0.8\;\mathrm{mm}$) to distinguish roughness from longer-wavelength waviness and form, in accordance with the ISO filtering standard (ISO 16610-21 [22]).

### 2.6. Dimensional Accuracy and Edge Sharpness

The dimensions of each sample were measured by a digital caliper with a nominal resolution of 0.01 mm. For each cube, measurements were taken at three locations along each axis, and the mean value was reported to account for local variations. To quantify edge sharpness (edge rounding), specimens were digitized using a 3Shape D800 scanner (3Shape, Copenhagen, Denmark). Since the printed parts were optically transparent, an AESUB scanning spray was applied as a thin matte coating to improve optical contrast and reduce surface reflections before scanning. Four cross-sectional slices were selected from the resulting scan, and edge sharpness was quantified using ImageJ (v1.8.0). The corner radius was computed by fitting a circle to the segmented corner contour in each cross-sectional slice. Nominal corner radius in CAD = 0 mm (sharp corner).

### 2.7. MTF Measurement

To quantify the optical performance of the projection system for both the 4K and 1080P projectors, the modulation transfer function (MTF) was measured at multiple defocus planes. An ISO 12233 slanted-edge target with an edge angle of 10° was projected and captured with a CMOS camera [23]. The recorded images were processed using a custom Python (v3.9.1) script to compute MTF curves from the slanted-edge method.

## 3. Results and Discussion

### 3.1. Polymerization Time

Table 1 presents the results of the polymerization start time induced by the applied illumination conditions and the selected projection times for each power level. The initiation of polymerization was identified by a slight change in refractive index, which was detectable in the shadowgraphy recordings and corresponded to the onset of the polymerization process. For each condition, the polymerization start time was determined from the first observable appearance of this refractive-index contrast, and the corresponding time was used as a reference point for defining subsequent projection intervals.

As shown in Table 1, increasing the Vis power reduced the polymerization start time. This time reduction was mainly led by the kinetics of free-radical photopolymerization. A higher Vis illumination power delivers a greater photon flux to the photoinitiators, accelerating the generation rate of free radicals. This rapid radical production consumes dissolved oxygen in the resin (which acts as a primary natural inhibitor) and accelerates the formation of the polymer network [4]. Conversely, the introduction of UV illumination did not lead to a measurable change in the polymerization start time under the investigated conditions. This result shows that during the earliest stages of the print, the start of gelation is dominated by the Vis-driven oxygen depletion rate. For further investigation, parts were removed from the resin container at three experimentally selected time intervals to evaluate the effect of total projection time on print quality, including dimensional accuracy and surface quality. In Table 1, the 1st, 2nd, and 3rd stop denote the cumulative projection time after the onset of polymerization (i.e., measured relative to the polymerization start time), and were chosen to capture early, intermediate, and extended exposure states for comparison.

### 3.2. Dimensional Analysis of the Printed Cubes

**Figure 2:**
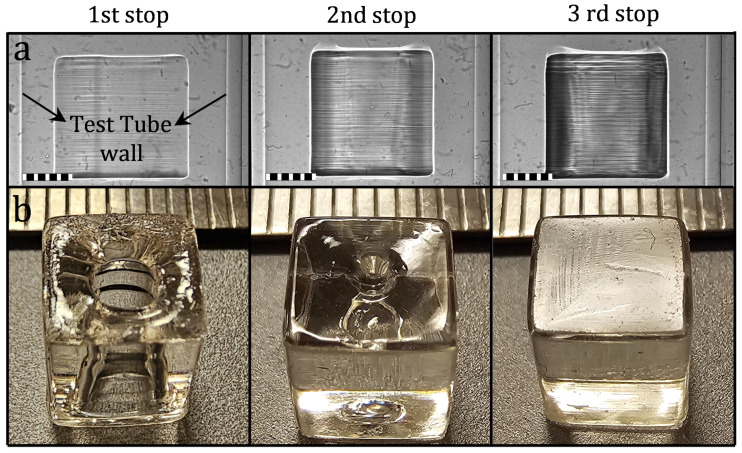
(**a**) In-situ shadowgraph of the printed cubes at different time intervals and 60% Vis power. Test tube walls are visible in the figure (scale bar: 3 mm). (**b**) Printed parts obtained after post processing.

Figure 2a illustrates the captured images of the 4*f* shadowgraph setup during the printing process at different time intervals, and Figure 2b shows representative printed parts. The observations show an “outside-in” polymerization mechanism in which polymerization initiates at the boundary of the cube and continues toward the center. In the early stages, the outer shell reaches the polymerization threshold first, forming a mechanically stable structure while an uncured core remains inside (Figure 2b, 1st and 2nd stops). With continued projection, the accumulated dose increases, and polymerization progresses through the center until a fully cured part is obtained (3rd stop). Observed edge-to-center threshold crossing is consistent with the non-uniform dose distribution expected in VAM when light propagates through an absorbing photopolymer. In a way that attenuation reduces the energy with increasing optical path and shifts the threshold crossing toward regions with shorter optical paths [7,8,9].

The thickness of the initially formed shell, and the balance between incomplete core curing and surface overexposure are sensitive to the effective optical attenuation of the resin. Attenuation is strongly linked to the photoinitiator (absorption spectrum and concentration). Adjusting the photoinitiator concentration provides a practical parameter to tune the polymerization threshold [7,15]. Increasing the photoinitiator concentration typically increases absorption and decreases the penetration depth, further shifting polymerization toward the edges. By changing the formulation of the resin, re-optimization of exposure conditions (such as, projection time and intensity, and, in dual-wavelength methods, the relative initiation/inhibition dose) will be necessary to restore comparable threshold behavior and dimensional fidelity [18,20,21].

This becomes more critical for larger test tubes, where the longer optical path reduces the delivered dose to the center. As a result, photoinitiator concentrations that were acceptable in smaller test tubes can promote sidewall polymerization and edge rounding unless compensated for by adjusting the light dosage and/or adding an inhibitor [7,8,9]. At last, in our samples, even after shell formation, scattering and absorption within the partially cured regions can lead to delivering an excess dose to the unwanted regions, driving over-polymerization that affects edges and diminishes dimensional accuracy (Figure 2b) [8,9].

**Figure 3:**
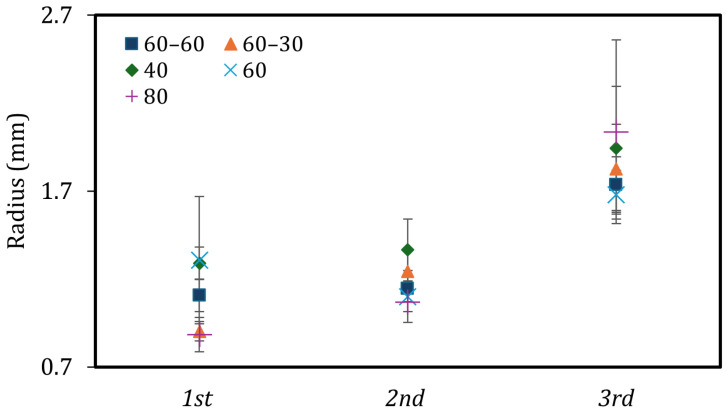
Corner radius of the printed cubes at different stops and projection powers.

Figure 3 summarizes the corner radius of the printed parts. As described previously, the outer shell of the part is initially generated by the attenuation-driven “outside-in” polymerization mechanism. A clear trend is detectable that as printing time increases, the corner radius increases. At the first stop, the initial shell formation preserves the intended sharp corners. However, for the next stops and higher illuminations, more energy is delivered to unwanted regions due to scattering and absorption in partially cured regions. This dose drives over-polymerization and lateral growth of the cubes. As a result, the sharp corners achieved at the 1st stop are rounded out and exhibit a larger radius at later stops. This loss of edge sharpness is more noticeable at higher light intensities, as the increased projection power rapidly accelerates over-polymerization.

In addition, measurable variations in corner geometry were recorded across each sample. This can be explained by the non-integer rotation of the vial during the printing. The projections were terminated at fixed time intervals, and the total illumination did not always match with full 360° rotations. As a result, certain regions received a lower dose than the rest of the geometry. This non-uniformity interrupts the symmetry of polymerization. Finally, to achieve better parts, the illumination and exposure times should be optimized so that part formation completes over an integer number of full rotations.

**Figure 4:**
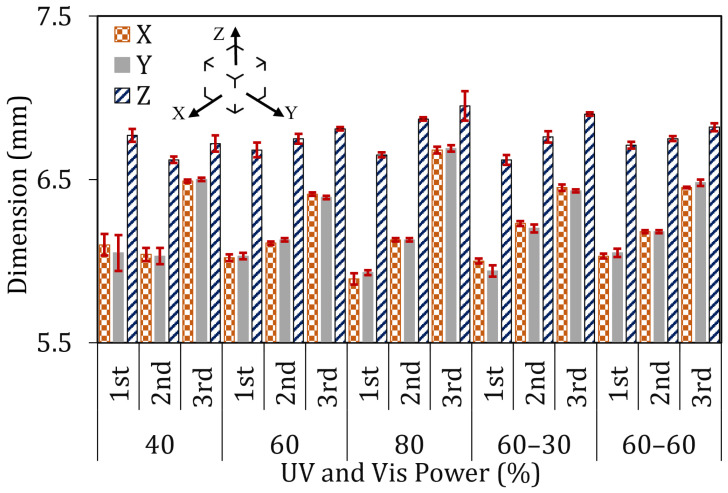
Dimensions of the printed parts at varying output powers (1st, 2nd and 3rd are corresponding to the samples at different termination times).

Figure 4 shows the measured dimensions of printed parts under different light engine power, with standard deviations calculated from three measurements per axis. According to the results, samples printed with 40% visible light power showed mean dimensions of 6.10 mm, 6.05 mm, and 6.77 mm along the *X*, *Y*, and *Z* axes, respectively. However, samples printed with longer projection times showed increased dimensions, aligned with over-polymerization, with recorded values ranging from 6.05 mm to 6.50 mm for the second and third termination times. A comparable behaviour was observed at 60% and 80% visible light power, showing that extended exposure results in dimensional growth beyond the intended geometry and reduces dimensional control. For example, at the third termination time, parts printed with 60% visible light power measured 6.41 mm and 6.39 mm along the *X* and *Y* axes, respectively. Higher values were recorded for 80% visible light power, reaching 6.69 mm and 6.68 mm. These results highlight the effect of higher power and longer projection time on dimensional accuracy.

Samples printed using 60% visible (Vis) light power had the lowest dimensional deviation. At the first termination time, the measured dimensions were 6.02 mm and 6.03 mm along the *X* and *Y* axes, respectively. These results show that, by careful optimization, the process can print accurate parts with optimized printing parameters. With the implementation of UV light at the same Vis engine power, a slight deviation from the nominal value was observed at the first termination time, which is attributed to the inhibition effect of UV illumination (Figure 4) [18].

A larger deviation was observed in the *Z*-direction, which is attributed to part sinking in the polymer vat. Part sinking in VAM might happen due to increased cured part density and the influence of gravity [24]. UV projections showed no significant effect on dimensional accuracy at longer projection times and could not prevent over-polymerization. Moreover, as evident from the projection patterns (Figure A1), UV illumination is confined to limited regions of the build volume; therefore, any inhibition it induces is spatially localized to those projected areas and may be too small in extent to counteract over-polymerization in the printed geometry.

**Figure 5:**
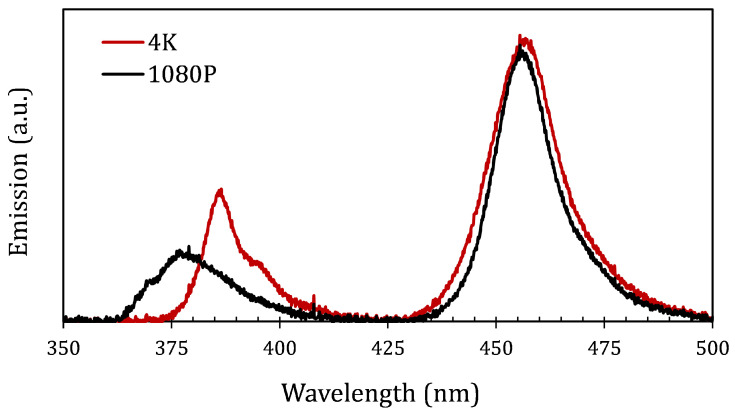
Emission spectrum of the projectors for both the 4K and 1080P.

The emission spectra of both the 4K and 1080P projectors, measured when both UV and Vis light sources were on, are presented in Figure 5. The two maxima observed in each curve correspond to the simultaneous projection of the distinct UV and Vis LED light engines within each projection system. The difference in UV between the projectors is because the 4K system uses a nominal 385 nm UV LED, whereas the 1080P system has a 365 nm UV LED. Furthermore, the spectrum shows that, for all the light engines, the projected wavelength bands are broader than their nominal values. For instance, the UV emitted by the 4K projector spans a broader spectral range, extending into the 410 nm region. Since the CQ/EDAB is also sensitive to wavelengths within this range, the UV illumination also triggers radical generation (initiation) alongside the intended HABI activation (inhibition). This reaction can impair the targeted inhibition effect of the HABI and might make the UV inhibition ineffective at confining the cure under the tested conditions.

### 3.3. Surface Roughness

**Figure 6:**
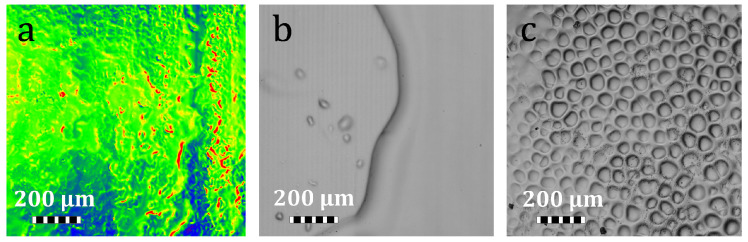
Effect of poor post-processing on the generated surface: (**a**) snakeskin-like surface morphology; (**b**) thin residual resin layer on the surface; (**c**) surface bubbles induced by ultrasonic washing.

PP is an essential step in VAM to improve and preserve the quality of the final printed part. Especially if high-viscosity resins have been used for printing, it can be difficult to clear from the printed geometry. Therefore, the PP procedure must be carefully tuned to the specific resin employed. Inappropriate PP, as defined here, is the incorrect selection of solvent, insufficient washing time, or resin and solvent temperatures, which fail to thoroughly remove uncured resin. This residual material affects the generated surface and may compromise performance, particularly for small features and fine edges that are more sensitive to residual resin and over-polymerization. As Figure 6 shows, an incorrect selection of PP procedures can lead to parts with defective surfaces and increased part-to-part variation. Also, it may form a snakeskin-like surface (Figure 6a), indicating non-uniform resin removal and local surface deformation. Furthermore, inadequate washing parameters can leave a thin residual resin layer on the surface. While this unwashed resin might misleadingly appear as a flat, successfully generated surface, it later cures during final illumination and changes the surface texture and dimensions (Figure 6b). Application of ultrasound during the IPA washing step also generated surface bubbles (Figure 6c). All these defects are not detectable by visual inspection and may only become visible during microscopic observation. Conversely, correctly selected PP procedure ensures complete dissolution of the uncured resin; as we applied here, that involved heating the test tubes to 60 °C for part release, a 10-min heated IPA wash, subsequent fresh IPA rinses, and final curing under a nitrogen atmosphere (Appendix A.3).

**Figure 7:**
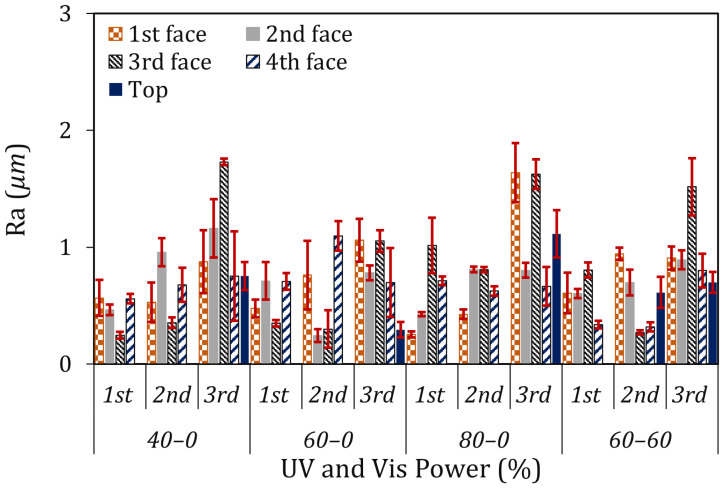
Surface roughness (*Ra*) of the printed parts with different light engine output power (1st, 2nd and 3rd are corresponding to the samples at different times).

Figure 7 demonstrates the surface roughness ($R_{a}$) of the printed parts. Measurements were performed on all faces except the face in contact with the plate during the PP step, to avoid artifacts introduced by contact and handling. The $R_{a}$ values (in μm) provide insight into how illumination settings affect the surface quality of the final printed parts and the degree of surface characteristic preservation. At 40% Vis power, the surface roughness of the first-stop samples ranged from 0.247 μm to 0.565 μm, increasing to 1.73 μm at the longest projection time (3rd stop). An increase in $R_{a}$ with higher Vis power was observed. Parts printed at 80% Vis power showed higher roughness than the parts printed at 60% Vis power, which indicated that higher exposure intensity promotes surface irregularities. In addition, surface roughness increased with longer projection times across all power levels (Figure 7), in a way that extended exposure increases the formation of surface asperities and accentuates variations from the intended surface finish.

The connection between light engine power and surface roughness is due to the polymerization process. Higher projection power accelerates polymerization and reduces process control, which leads to rough surfaces. These findings highlight the necessity of projection power and exposure time optimization to achieve better surface quality and maintain dimensional accuracy.

**Figure 8:**
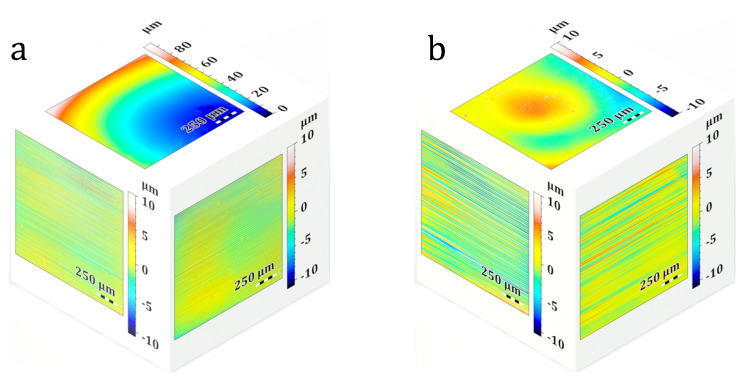
Surface scan of the printed cubes at different visible (Vis) light power settings: (**a**) 40% and (**b**) 60% (scale bar: 250 μm).

Figure 8 shows representative confocal microscopy images of the generated surfaces. As shown in the figure, a uniform pattern of horizontal lines is distributed across the measured area. The spacing (pitch) of these lines was approximately 25 μm. These features are likely related to spatial nonuniformities in illumination and the DMD pixel structure, which can be imprinted on the printed surface. In other words, small intensity deviations in the projected image can translate into local differences in cure depth and surface height, producing a repeatable, periodic surface texture [25,26]. While this pattern is consistently observed across all samples, the comparison between the 40% and 60% VIS light power settings (Figure 8a vs. Figure 8b) shows a difference in surface roughness. The increased power amplifies the overall surface roughness because the accelerated polymerization at higher intensities more prominently captures and creates these minor optical fluctuations.

**Figure 9:**
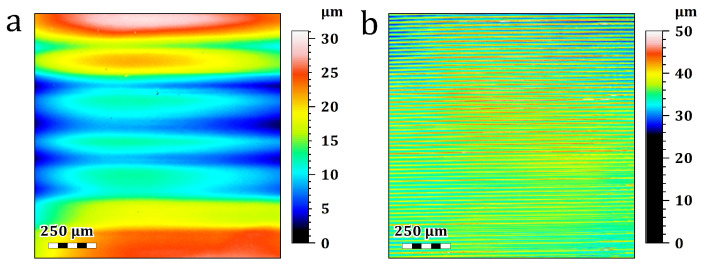
Surface scan of the printed parts obtained using (**a**) the 1080P projector and (**b**) the 4K projector.

**Figure 10:**
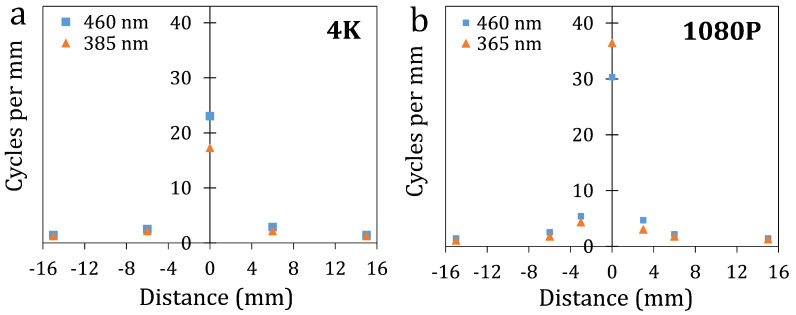
MTF50 spatial frequency as a function of distance (defocus) for the (**a**) 4K projector and (**b**) 1080P projector at the indicated wavelengths.

To physically verify that these surface patterns are directly connected to the light source, new samples were printed using a lower-resolution projector (1080P) and re-evaluated. To isolate the effect of the optical system, identical printing parameters were used for both setups. In the 4K projector setup, the focal plane was positioned 6 mm away from the test tube center. For the 1080P projector, the focal plane was aligned exactly with the center of the tube. As shown in Figure 9, the linear pattern is still visible. However, the spacing increased to approximately 150 μm. The larger effective pixel size and the lower spatial sampling of the projected image in the lower-resolution system can justify this increase. Thus, the observed surface pattern is directly linked to the projector resolution, and the measured 150 μm pitch aligns exactly with the resolvable feature size at the surface position of the printed part (which will be further correlated with the MTF analysis in Figure 10).

The projectors are focused at a specific position and achieve the best optical resolution only at the focal plane. Due to a limited depth-of-focus of 2–3 mm, image sharpness decreases as the projection moves farther into the print volume. To quantify this, MTF measurements were taken at various distances from the focal plane. This approach allowed us to correlate the spatial frequency of line patterns on the printed surface with that of the projected patterns at different planes within the test tube. Figure 10 shows the extracted ${\mathrm{MTF}}_{50}$ spatial frequencies for both projectors at multiple defocus planes along the print volume. The ${\mathrm{MTF}}_{50}$ spatial frequency was approximately 23 cycles/mm at 460 nm and 17 cycles/mm at 385 nm for the 4K projector in the focal plane. At a distance of 6 mm from the focal plane, the frequency decreased to 2.5 cycles/mm. For the 1080P projector, ${\mathrm{MTF}}_{50}$ spatial frequencies of ∼30 cycles/mm at 460 nm and ∼36 cycles/mm at 365 nm were obtained at the focal plane, while only a few cycles/mm were maintained at defocused planes.

The effective resolvable feature size at the focal plane of the 4K projector was approximately 23 μm. For the 1080P projector, the feature size was about 100 μm at 3 mm and 200 μm at 6 mm from the focal plane. This spatial variation denotes a key process limit. For instance, if the target print features are smaller than the resolvable spatial frequencies at a specific depth, the projector will be unable to accurately generate those features. By checking the cube’s outer-shell position within the test tube during printing, the resolved spatial frequencies can be linked to those ∼25 μm and ∼150 μm pattern pitches that we observed for the 4K and 1080P projectors, respectively.

Uncertainty sources in the VAM process include variations in the light engine, measurement errors, and the PP step. Any light engine power instability and optical alignment variations can lead to inconsistent polymerization across the test tube and between prints, which can easily affect both dimensional accuracy and surface quality. The precision and accuracy of the characterization instruments, such as calipers, microscopes, and confocal microscopes, contribute to measurement uncertainty. PP introduces further changes, since washing time, solvent condition, agitation/ultrasonic conditions, drying, and post-curing can each influence residual resin removal and the final degree of cure. Finally, these factors impact the overall quality and reliability of VAM printed parts.

## 4. Conclusions

In this study, the dimensional fidelity and surface quality of the VAM printed parts were evaluated as a function of light engine power for UV and Vis projections. Cubic parts were successfully printed, with the best accuracy obtained at the first termination time with a maximum lateral variation of 1.8%. Polymerization initiation time was reduced by accelerating free radical generation and oxygen depletion via higher Vis power. On the other hand, increasing the projected Vis dose by higher intensities and/or longer projection times narrows the process window and can easily induce over-polymerization due to light scattering and absorption in partially polymerized regions. These characteristics resulted in dimensional variations beyond the desired geometry, such as increased corner radius and higher surface roughness. However, under the tested conditions, UV projections did not produce a measurable change in the printed parts, and Vis exposure dominated the polymerization process. This effect was linked to the broad emission spectra of the projectors, which can trigger the primary photoinitiator alongside the inhibitor.

Confocal microscopy showed a periodic line texture on printed surfaces. Spatial-frequency evaluation across defocus planes showed that the resolvable spatial frequency at print locations matched the measured patterns. Face-to-face variability in the printed parts was caused by irregular projections, such as non-integer rotation numbers. These irregularities caused a non-uniform dose distribution. This study demonstrated the importance of stable dose delivery, controlled projection conditions, and standard PP to achieve reproducible, high-fidelity VAM parts.

## Figures and Tables

**Table 1:** Polymerization start and selected projection times (shortest projection time is at the 1st stop and longest projection is the 3rd stop).

Vis–UV Power (%)	Polymerization Start Time (s)	1st Stop (s)	2nd Stop (s)	3rd Stop (s)
40	100	20	25	30
60	70	15	20	25
80	52	10	15	20
60–30	70	15	20	25
60–60	70	15	20	25

**Figure A2:**
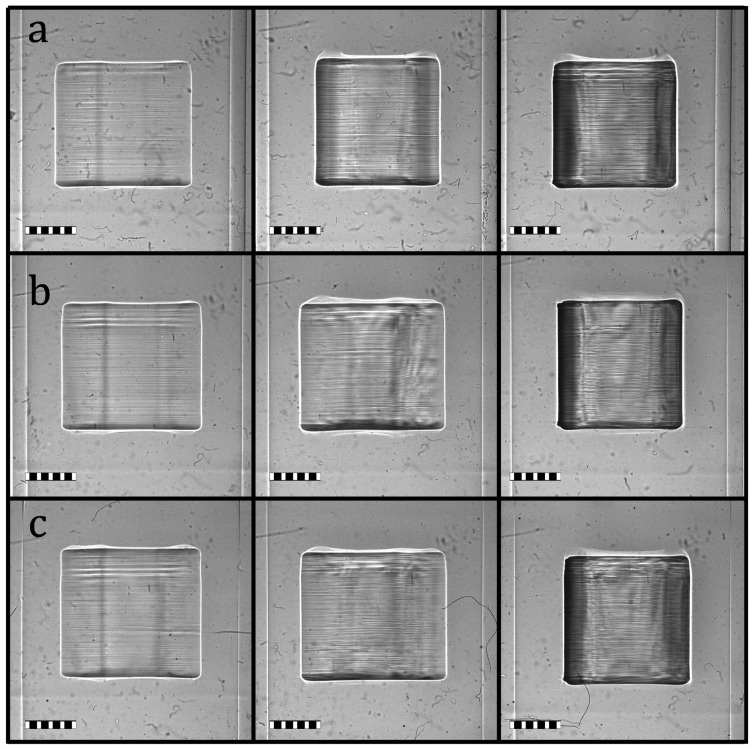
In-situ shadowgraphy of the printed cubes at different time intervals under three Vis–UV projection power settings: (**a**) 60–0%, (**b**) 60–30%, and (**c**) 60–60% (scale bar: 3 mm).

## Data Availability

The original contributions presented in this study are included in the article. Further inquiries can be directed to the corresponding author.

## Abbreviations

The following abbreviations are used in this manuscript:

AM	Additive Manufacturing
VAM	volumetric additive manufacturing
CAL	Computed Axial Lithography
Vis	Visible (light)
UV	Ultraviolet (light)
DMD	Digital Micromirror Device
MTF	Modulation Transfer Function
CAD	Computer-Aided Design
fps	Frames per second
TEGDMA	Triethylene glycol dimethacrylate
BisGMA	Bisphenol A glycerolate dimethacrylate
CQ	Camphorquinone
EDAB	Ethyl 4-dimethylaminobenzoate
o-Cl-HABI	2,2^′^-Bis(2-chlorophenyl)-4,4^′^,5,5^′^-tetraphenyl-1,2^′^-biimidazole
THF	Tetrahydrofuran

## Appendix A

### Appendix A.1

To illustrate the tomographic input data, Figure A1 shows representative 2D projection patterns for the visible (Vis) and ultraviolet (UV) illumination channels. The green and red color channels represent the Vis and UV projection frames, respectively.

### Appendix A.2

In the main text, Figure 2 shows representative in-situ shadowgraphy acquired at 60% Vis power. For completeness, Figure A2 provides the corresponding shadowgraphy sequences for three printing conditions.

### Appendix A.3

Design of experiments (DOE) screening was performed to select a reliable PP procedure to minimize surface defects (e.g., residual resin film, surface bubbles, or snakeskin-like morphology) and yield tack-free (non-sticky) surfaces. The DOE matrix and the surface quality for each experiment are listed in Table A1. Surface state was evaluated qualitatively and supported by confocal microscopy. To show how the PP parameters affect the surface condition, representative confocal microscopy scans from two DOE runs are shown in Figure A3, corresponding to the best and worst outcomes. In the best condition (Figure A3a), the solvent washing step removed the majority of the uncured resin from the surface, enabling observation of the underlying patterns. However, a small amount of residual resin remained on the surface. This residual material is attributed to the absence of an additional rinsing step during the DOE screening, which was later added in the finalized PP protocol used for the main samples in this study. In contrast, the worst condition (Figure A3b) appears macroscopically smooth and nearly defect-free in the scan, yet the sample was identified as a failed part because unwashed resin remained on the surface. During the illumination, this residual resin polymerized and formed a continuous layer that became integrated into the part. This remaining cured layer effectively misleads the true representative of the surface, despite it might seem to be a perfect flat surface.

**Table A1:** Design-of-experiments (DOE) matrix used for post-processing parameter screening.

Sample	Resin Temp. (°C)	Solvent Temp. (°C)	Wash Time (min)	Environment	Intensity (mW/cm^2^)	PP Time (min)
1	RT	60	1	N_2_	5	5
2	RT	60	10	N_2_	5	1
3	60	RT	10	O_2_	80	1
4	60	60	1	O_2_	5	1
5	60	RT	1	O_2_	80	5
6	RT	RT	10	O_2_	5	1
7	60	60	10	N_2_	80	1
8	60	60	10	O_2_	5	5
9	60	RT	1	N_2_	5	1
10	60	60	1	N_2_	80	5
11	RT	RT	1	O_2_	5	5
12	RT	60	1	O_2_	80	1
13	RT	60	10	O_2_	80	5
14	60	RT	10	N_2_	5	5
15	RT	RT	10	N_2_	80	5
16	RT	RT	1	N_2_	80	1

RT denotes room temperature. All tack-free outcomes were obtained under N_2_.

## References

[1] Wang X., Ma Y., Niu Y., Xiong B., Zhang A., Zhang G., Chen Y., Wei W., Fang L., Wu J. (2026). Sub-second volumetric 3D printing by synthesis of holographic light fields. Nature.

[2] Álvarez-Castaño M.I., Madsen A.G., Madrid-Wolff J., Sgarminato V., Boniface A., Glückstad J., Moser C. (2025). Holographic tomographic volumetric additive manufacturing. Nat. Commun..

[3] Whyte D.J., Doeven E.H., Sutti A., Kouzani A.Z., Adams S.D. (2024). Volumetric additive manufacturing: A new frontier in layer-less 3D printing. Addit. Manuf..

[4] Thijssen Q., Toombs J., Li C.C., Taylor H., Van Vlierberghe S. (2023). From pixels to voxels: A mechanistic perspective on volumetric 3D-printing. Prog. Polym. Sci..

[5] Madrid-Wolff J., Toombs J., Rizzo R., Bernal P.N., Porcincula D., Walton R., Wang B., Kotz-Helmer F., Yang Y., Kaplan D. (2023). A review of materials used in tomographic volumetric additive manufacturing. MRS Commun..

[6] Shusteff M., Browar A.E.M., Kelly B.E., Henriksson J., Weisgraber T.H., Panas R.M., Fang N.X., Spadaccini C.M. (2017). One-step volumetric additive manufacturing of complex polymer structures. Sci. Adv..

[7] Kelly B.E., Bhattacharya I., Heidari H., Shusteff M., Spadaccini C.M., Taylor H.K. (2019). Volumetric additive manufacturing via tomographic reconstruction. Science.

[8] Bhattacharya I., Toombs J., Taylor H. (2021). High fidelity volumetric additive manufacturing. Addit. Manuf..

[9] Loterie D., Delrot P., Moser C. (2020). High-resolution tomographic volumetric additive manufacturing. Nat. Commun..

[10] Regehly M., Garmshausen Y., Reuter M., König N.F., Israel E., Kelly D.P., Chou C., Koch K., Asfari B., Hecht S. (2020). Xolography for linear volumetric 3D printing. Nature.

[11] Rackson C.M., Toombs J.T., De Beer M.P., Cook C.C., Shusteff M., Taylor H.K., McLeod R.R. (2022). Latent image volumetric additive manufacturing. Opt. Lett..

[12] Rackson C.M., Champley K.M., Toombs J.T., Fong E.J., Bansal V., Taylor H.K., Shusteff M., McLeod R.R. (2021). Object-space optimization of tomographic reconstructions for additive manufacturing. Addit. Manuf..

[13] Orth A., Sampson K.L., Ting K., Boisvert J., Paquet C. (2021). Correcting ray distortion in tomographic additive manufacturing. Opt. Express.

[14] Orth A., Webber D., Zhang Y., Sampson K.L., de Haan H.W., Lacelle T., Lacelle T., Webber D., Fatehi D., Boisvert J. (2022). On-the-fly 3D metrology of volumetric additive manufacturing. Addit. Manuf..

[15] Cook C.C., Fong E.J., Schwartz J.J., Porcincula D.H., Kaczmarek A.C., Oakdale J.S., Moran B.D., Champley K.M., Rackson C.M., Muralidharan A. (2020). Highly tunable thiol–ene photoresins for volumetric additive manufacturing. Adv. Mater..

[16] Toombs J.T., Luitz M., Cook C.C., Jenne S., Li C.C., Rapp B.E., Kotz-Helmer F., Taylor H.K. (2022). Volumetric additive manufacturing of silica glass with microscale computed axial lithography. Science.

[17] Kollep M., Konstantinou G., Madrid-Wolff J., Boniface A., Hagelüken L., Sasikumar P.V.W., Blugan G., Delrot P., Loterie D., Brugger J. (2022). Tomographic volumetric additive manufacturing of silicon oxycarbide ceramics. Adv. Eng. Mater..

[18] Wang B., Engay E., Stubbe P.R., Moghaddam S.Z., Thormann E., Almdal K., Islam A., Yang Y. (2022). Stiffness control in dual color tomographic volumetric 3D printing. Nat. Commun..

[19] Wang B., Sun W., Safari H., Hedayati S.K., Narag J.P.C., Christiansen T.D.V., Schiefler A.A., Sørensen H.O., Frisvad J.R., Almdal K. (2023). Lateral Contrast Enhancement in Tomographic Volumetric 3D-Printing via Binary Photoinhibition. arXiv.

[20] van der Laan H.L., Burns M.A., Scott T.F. (2019). Volumetric photopolymerization confinement through dual-wavelength photoinitiation and photoinhibition. ACS Macro Lett..

[21] de Beer M.P., van der Laan H.L., Cole M.A., Whelan R.J., Burns M.A., Scott T.F. (2019). Rapid, continuous additive manufacturing by volumetric polymerization inhibition patterning. Sci. Adv..

[22] (2011). Geometrical Product Specifications (GPS)—Filtration—Part 21: Linear Profile Filters: Gaussian Filters.

[23] (2024). Digital Cameras—Resolution and Spatial Frequency Responses.

[24] König N.F., Reuter M., Reuß M., Kromer C.S.F., Herder M., Garmshausen Y., Asfari B., Israel E., Vasconcelos Lima L., Puvati N. (2025). Xolography for 3D Printing in Microgravity. Adv. Mater..

[25] Kang J.-W., Jeon J., Lee J.-Y., Jeon J.-H., Hong J. (2024). Surface-Wetting Characteristics of DLP-Based 3D Printing Outcomes under Various Printing Conditions for Microfluidic Device Fabrication. Micromachines.

[26] Caplins B.W., Higgins C.I., Kolibaba T.J., Arp U., Miller C.C., Poster D.L., Zarobila C.J., Zong Y., Killgore J.P. (2023). Characterizing light engine uniformity and its influence on liquid crystal display based vat photopolymerization printing. Addit. Manuf..

